# Pathogenesis, Diagnosis, Antimicrobial Therapy, and Management of Infective Endocarditis, and Its Complications

**DOI:** 10.7759/cureus.29182

**Published:** 2022-09-15

**Authors:** Saakshi P Kamde, Anil Anjankar

**Affiliations:** 1 Pathology, Jawaharlal Nehru Medical College, Datta Meghe Institute of Medical Sciences, Wardha, IND; 2 Forensic Medicine, Jawaharlal Nehru Medical College, Datta Meghe Institute of Medical Sciences, Wardha, IND

**Keywords:** treatment management, eosinophilic zone, inflammatory, health care, antimicrobial therapy, diagnosis, pathologies, infective endocarditis

## Abstract

Infective endocarditis in the adult is life-threatening. Bacterial endocarditis is an inner infection lining the heart muscle (endocardium). The scientific study of the causes of diseases is known as etiology. The agents that cause disease fall into five groups: bacteria, viruses, protozoa, fungi, and helminths (worms). Risk factors are past heart defects, damaged or abnormal heart valves, new valves after surgery, chronic hemodialysis, and immunosuppressed state (chemotherapy, HIV, etc.). Infective endocarditis is categorized into two clinical forms: bacterial acute and subacute endocarditis. Acute bacterial endocarditis is usually caused by *staphylococci (staph)* and *streptococci (strep)*. And occasionally by *listeria *and *brucella* bacterial strains. Invasive medical technology has increased the responsibility of healthcare-associated infective endocarditis (HAIE). Microscopy of the disease is the chronic aggressive cells in the deeper zone of nonspecific, composed of fibrin and platelets covering colonies of bacteria. Tuberculous valvular endocarditis due to mycobacterium tuberculosis is a rare clinical entity. Syphilitic endocarditis is pathologically the cutaneous lesions of secondary syphilis. It is caused by infection with the microorganism*Treponema pallidum*. Fungal endocarditis is a rare and fatal condition. They are infected with fungi such as *Candida albicans, Histoplasma capsulatum*, and *Aspergillus* species. Fatal endocarditis associated with Q fever (query fever). Q fever is a chronic or prolonged disease caused by the *rickettsial*-like *bacillus Coxiella burnetii*, a rare form of *rickettsia* in the endocarditis. Varicella-zoster virus (VZV) infection causes chronic and repeated febrile illness. They are followed by pharyngitis, malaise, and a vesicular rash. Chronic Q fever usually manifests as endocarditis or hepatitis. The therapy given to simplify the complications is antimicrobial therapy. The medicines prescribed are ampicillin, cefazolin, ceftazidime, gentamicin, vancomycin, metronidazole, and tobramycin. High medicinal antibiotics are used to control the spread of infective endocarditis.

## Introduction and background

Infective endocarditis is a potentially deadly disease affecting the host and pathogen changes. The epidemiology of infective endocarditis has become tangled with healthcare-associated factors predisposing to infection. Mitral stenosis is a narrowing of the heart's mitral valve. The valve doesn't open properly, blocking blood flow into the main pumping chamber of your heart (left ventricle). Mitral valve stenosis can make the person tired and short of breath. The pathophysiology comprises three critical elements: preparation of the cardiac valve for bacterial adherence, adhesion of circulating bacteria to the prepared valvular surface, and survival of the adherent bacteria on the surface with the propagation of the infected vegetation [1,2]. Under usual conditions, fungal infections are rare. The immune system protects against these infections. The risk is due to intravenous drugs, pacemakers, and cognitive heart diseases of artificial valves. The ultrasound of the heart or blood cultures are the supported tests and suspected symptoms. Patients with pacemaker leads, artificial heart valves, and abnormal heart valves are more affected. The developing infective endocarditis can predispose to structural heart disease [3-5]. A life-threatening illness deals with mobility and mortality. The use of antibiotic medical care has weakened the death rate of valve endocarditis [6].

Infective endocarditis vegetations grow on the valves and produce toxins and enzymes that kill and break down the tissue to cause holes in the valve. The resulting complications are: Embolism of material from the vegetation can get in the way of blood flow. Valve replacement therapy and intra-cardiac instruments are increased in hospitalization. Diabetes, organ transplants, end-stage renal disease, and human immunodeficiency syndrome are increased, which are the dominant factors of chronic diseases. The most commonly affected region is the endocardial lining, which causes deadly diseases worldwide. The relevant risk factors in any unhealthy individual can have different diagnostic aspects [7,8].

Cardiac severe diseases are mainly due to bacterial infection. Rheumatic fever and endocarditis are two examples. Certain blood-borne bacteria come in contact with the endocardial lining and cause bacterial endocarditis. The anatomic cardiac defect and abnormal valve are mainly considered. There are two types of infective endocarditis: subacute and acute [9]. The virulence of the organism decides whether the infection is acute or subacute. The organism with more virulence is called subacute, and low virulence is called an acute infection. The significant feature difference between subacute and acute is shown in table 1. Resistance to the scarce new antibiotics is also emerging. It would help to optimize the armamentarium of antibiotics to preserve new antibiotics and avoid the prescription of molecules known to favor the spread of resistance.

**Table 1:** Feature differences between subacute infective endocarditis and acute infective endocarditis

Features	Subacute	Acute
Clinical features	Splenomegaly, clubbing of fingers, petechial	Acute systemic infection
Lesions on valves	Not invasive, suppurative	Invasive, damaging, suppurative
Previous condition	Previously damaged	Usually previously normal
Virulence	Less virulent	Highly virulent
Most common organism	Streptococcus viridians	Staphylococcus aureus
Duration	> 6 weeks	< 6 weeks

## Review

Pathogenesis

Pathogenic bacteria find ways to enter and exit the bloodstream and the surrounding tissues through the endothelium. It is an interface between circulating blood or lymph in the lumen and the rest of the vessel wall [10,11]. Platelets, fibrin deposition, and surface disruption may lead to valve trauma and produce endothelial cell alternation. The surface is susceptible to colonization by the circulating bacteria, which render and do not cause any harm. Fibrin platelet matrix is more common than others; some bacterial strains appear to adhere most quickly to the surface lining. The complex, which attaches to the lining, is most commonly polysaccharide (dextran), which affects the virulence factor of the bacteria. The surface vegetation's complex, and the adherent bacteria survive on the endothelial surface. The clotting cascade is initiated by vegetative propagation. Bacteria and fungi infections usually do not affect the standard endothelial lining of the heart and its valves. Vegetation on the endocardium and a sequence of interrelated consequences occur before microbes are tested. The infecting organism of the vegetative formation depends on pathogenesis.

Vegetation formation is a multistep process. The first step is endocardial injury. The most common mechanism is injury by turbulent blood movement from congenital intracardiac abnormality. The microorganisms circulate the blood sterile platelet and fibrin formation. The dermal and the mucosal are infections of transient bacteremia. The extrinsic clotting pathway of the coagulation system is activated further, which causes the release of various cytokines from the adherent monocytes. Further deposition of fibronectin continues as the endothelial cells start. Vegetation is the culmination of a microscopic event [12,13]. The ongoing infection becomes more difficult to eradicate bacteria's growth inside the fibronectin matrix nectin, making the host's immune system not respond appropriately. *Staphylococcus aureus (S. aureus)* is a species with high virulence and can infect the standard cardiac valves of a human being. Subsequent internalization into the endothelial cells and the extracellular matrix binding protein are mechanisms that bind the *S. aureus* bacterium [14]. They further induce tissue destruction by releasing exoproteins, which endothelial cells activate when the bacteria invade the host's immune system. 

Myocardial abscesses, tissue destruction, conduction system abnormalities, and sudden spots cause heart failure or death of the human. These are the exact consequences of local infection. The involvement of valve ring abscesses is due to a wide spread of diseases of prosthetic valve infections, particularly aortitis. The valve obstruction is manifested by mycotic aneurysms, myocardial abscesses, obstructing vegetation conduction disturbances, and dehiscence. Systemic chronic illness is embolizing infected substances from the heart valve immune-medicated phenomena. Septic pulmonary embolus is a right-sided lesion that causes empyema, pneumonia, and pulmonary diseases. The left-handed lesion embolizes the central nervous system, spleen, kidney, and other tissues [15]. Diffuse glomerulonephritis frequently occurs in retinal emboli, cutaneous, and deposition. The endothelial cells produce any alteration if any trauma occurs to the valves.

The critical damages are possible in infective endocarditis pathogenesis. Including bacteriemia, polycythemia, cyanosis, endothelial damage, and increased blood viscosity are leading to complications. The most noticeable increase is in artificial implants such as prosthetic valves and shunts. It is not astonishing that cyanotic CHD with an unreal shunt or prosthetic valves represents the highest risk for infected endocarditis. Neonatal endocarditis often occurs on the heart's right side and is associated with disruption of the endocardium or valvular endothelial tissue produced by catheter-induced trauma [16]. The dermal and mucous membranes experience frequent trauma due to bacteria in premature neonates or the placement of umbilical or peripheral venous catheters, parenteral hyperalimentation, and vigorous endotracheal suctioning. The bacteremia combination and the endothelial damage are the critical induction of infective endocarditis [17].

Infective endocarditis diagnosis and role of blood culture

Various diagnostic tests are performed for infective endocarditis. With the help of symptoms, treating the patient well and diagnosing the disease is easier. Medical tests and symptoms are important factors. Tests that help to detect endocarditis are blood culture tests, which help in detecting the germs in the blood. The combination of the antibiotics and treatment usually depends on the blood test report. The complete blood count appearance increases in the white blood cells. A blood culture test result helps to find infection in the bloodstream. Septicemia is an infection that occurs when bacteria enter the bloodstream. It can lead to sepsis, the body's reaction to the disease, which can cause organ damage and even death, a sign of endocarditis [18].

Further blood tests other than these are carried out to check the extent of the infection. In an echocardiogram, the sound waves create images of the heart's beating. It is the test that shows the heart chambers and the blood pumping through the heart's valves. The structure of the heart indicates in this test. Two types of echocardiograms are used in the medical field. In a transthoracic (standard) echocardiogram, a wand-like device is moved over the chest area. A transesophageal echocardiogram guides a tube that connects the throat to the stomach. It provides more details about the heart structure. The electric activity measured by this method is usually painless and quick. The leads of ECG are generally attached to the arms and legs also. It can show the electrical activity of the heart if anything happens. It is not used explicitly for the detection of endocarditis. Another test is the chest X-ray, which helps to see the internal organs, like the heart and the lungs. If any infection has spread in the lungs or any swelling in the gut, it can be seen in the X-ray. The two other tests performed are MRI and CT scan. These tests are mostly done to scan the brain, chest, or other body parts if the infection has spread.

The infecting microorganisms in the blood are reflected in the fatal disease of endovascular endocarditis. Determining the disease condition and blood culture is the primary test for the microbiologic etiologic. Routine hematological cultures procreate on automated observation for almost all endocarditis cultivable agents. This process helps to avoid accumulative extended incubation or last subculture. Guidelines are set for the timing and the number of blood cultures as recommended. According to the European Society of Cardiology and the American Heart Association, the different venepuncture sites are the three sets of blood cultures. There should be a one-hour gap between the first and the last. In the subacute, chronic endocarditis, three blood samples and acute sepsis are collected in one hour to six hours. The British Society for Antimicrobial Chemotherapy (BSAC) recommends antimicrobial chemotherapy.

One anaerobic and one aerobic bottle contains three sets of blood cultures. They are gathered in two groups of bottles, two oxidative and one anaerobic bottles per set (six bottles in total). The adequately filled bottle and the yield of the culture are directly proportional to the volume of the blood culture. Most blood cultures of a long-suffering patient with endocarditis originate from organisms cultivated in the culture systems blood must be affirmative. The benefit of collected blood culture supply is that blood tastes are suitably gathered and drawn preceding the management of antimicrobial medical aid and individual. The correspondence to an endocarditis pathogen should typically show the positive culture of the blood sample. Though the theory of attributed arrangement of never-ending changes in bacteremia was published in the referred direction, modification of culture of blood draws over instance is not the statistic for routine blood culture [19]. We are unaware of evidence supporting the value of spaced blood culture draws for etiologic diagnosis of endocarditis. Routinely spacing blood culture examination is not recommended in cases of suspected endocarditis. Standard blood culture with five days of incubation time is adequate for healing almost all cultivable causes of endocarditis, including *Candida* species.

The organisms *Aggregatibacter, Haemophilus, Cardiobacterium, Eikenella, and Kinsella* are challenging to discover in the blood cultures due to their delicate nature. Earlier the extended incubation periods were considered with existing blood culture systems, prolonged incubation (and end blind subculture) redundant for the recovery of the organisms, as they are well grown and noticed within the regular five-day incubation period. The actual blood culture method also contains sufficient slenderness to support the growth of *abiotrophia* and *granulicatella* species. *Brucella* species occasionally cause endocarditis and are observed in routine blood cultures. The non-serologic examination may be facilitative if the exposures indicate Brucella endocarditis. *Cutibacterium* (*Propionibacterium*) *acnes (C. acnes*) deserve detailed information. However, the species may require an extended blood culture incubation period. According to the Clinical and Laboratory Standards Institute guidelines, the end subculture of the chocolate medium is recommended if the other blood cultures show a negative report of endocarditis [20]. The usefulness of the blind subculture usually fails the shreds of evidence; The BSAC guidelines do not approve the practice.

The species grown in blood culture are *candida* species that cause fungal endocarditis. *Histoplasma capsulatum* and *aspergillus* species are rare non-*candida* fungi which cause endocarditis. Specialized fungal blood cultures and antigen detection are the testing methods for endocarditis. Many people with endocarditis are successfully treated with antibiotics. Sometimes, surgery is suggested to fix damaged heart valves and clean up the infection. The cultures must be collected appropriately. The most frequently occurring etiologies culture as negative endocarditis is *Coxiella burnetii* (*C. burnetii*). *Bartonella* species are tiny, gram-negative bacilli that cause endocarditis. *Bartonella quintana*, the agent of trench fever, and *Bartonella henselae*, the agent of cat-scratch disease, are the significant causes of Bartonella endocarditis. Due to the prolonged incubation period of strains and the bacterial species of the blood culture, *C. acnes* is an infrequent cause of endocarditis. The molecular diagnostic method of negative culture endocarditis is a unique factor considering mycoplasma pneumonia. An antibody serologic testing shows the immune system that can fight off certain diseases. Due to nonspecific clinical and radiographic presentations, community-acquired pneumonia (CAP) includes *Legionella*, *Chlamydophyla*, or *Mycoplasma* diagnostic challenges. Numerous microbiologic tools have been developed to determine intensive infection.

The traditional bacterial culture does not show any serological reports positively (e.g., *C. burnetii*) or in particular (e.g., *Bartonella* species). Diagnosis can aid the serological evaluation. Brucella endocarditis is caused by infection of *Brucella* species. However, according to modified Dukes criteria, these pathogens cause sub-acute endocarditis, which results in the elevation of IgG titters [21]. The best-accepted serologic effort to identify endocarditis is the serology of *C. burnetii*. According to modified Duke's criteria. However, most often diagnosed Bartonella endocarditis is the method of serologic testing. The etiological diagnosis of the infective endocarditis gets complicated by applying serological inter cross-reactivity. *Chlamydophila abortus (*C. abortus), a gram-negative intracellular bacterium, is the species responsible for enzootic abortion. *C. abortus* was previously identified as *Chlamydia psittaci*. Associate infections with *C. abortus* and *C. burnetii* are repeatedly reported, resulting in a false positive. The serologic testing causes rare endocarditis, which is why Oslerian manifestation is not recommended. The immunologic vascular, peripheral emboli, and active valvulitis are evidence of bacteremia or fungemia. The HACEK group of bacteria (*Haemophilus species*, *Aggregatibacter species*, *Cardiobacterium hominis*, *Eikenella corrodens*, and *Kingella species*) are a small, heterogeneous group of fastidious microorganisms that are an unusual cause of infective endocarditis. The left-sided valvular involvement does not create vascular phenomena and peripheral emboli. 

The variations in the clinical representation quality require the most critical planning. Patients in the IVDA group (intravenous drug abuse group) *S. aureus* with right-sided infective endocarditis were rejected as definite cases. The parameters vascular complications, new regurgitate murmurs, and persistent bacteremia are the diagnostic properties. More or less, the finding of echocardiographic help is now that IVDA is accepted as a progressively important inherent comorbid assumption for development. The high mortality rate is most common in right-sided infective endocarditis. Opposite the coronary sinus, the tricuspid valve is almost affected. The coronary sinus vegetation, the outcomes, the treatments, and the diagnosis are not yet summarized.

The abnormal laboratory test and the multitude of signs and symptoms can be the cause of endocarditis. A definite diagnosis is difficult because many are nonspecific [22]. The syndromic diagnosis, also called endocarditis, requires various observations and findings for the approval of the disease as it is multi-symptomatic. The disease diagnosis is based on a combination of laboratory and clinical findings. The patient can undergo surgery or autopsy if only accepted by the Beth Israel criteria. In practice, a trial by randomization of treatment seems likely to enable a reasonable decision. The process of analyzing a case should give different weights to different findings. The Jones criteria, used in acute rheumatic fever, are the better-known example of the weighting of diagnostic properties. It seems suitable to use a parallel approach toward disease to diagnose infective endocarditis. The Duke's criteria highly define minor and significant symptoms to apply to surgery if necessary. The subgroup of patients is made of the ones who are drug users with right-sided endocarditis patients with valve infections. Infants with congenital heart disease and complicated prosthetic valve infections are also a subgroup of the Dukes' criteria [23].

The diagnostic challenge for physicians is culture-negative endocarditis. Traditional methods such as histology, serology, and culture new molecular techniques have been formulated to improve the detection of complex culture agents. The serologic tests looked for antibodies in the blood. Serological tests focus on proteins the disease caused by etiologic agents, *Coxiella burnetii* and *Bartonella*. The use of novel tissue cell lines can reduce the sensitivity of the culture of bacteria which has been done by improved injection of the samples in the shell vials. If the patient has a positive blood culture and a cardiac valvular lesion, the diagnosis is straightforward on the first consideration. Probable panic situation is created when even a single suspected case is reported. A wide range of species can cause this: the direct visualization of the primary lesion in the valvular vegetation and different symptoms that may be seen as the disease. They can mimic any other illness which may have a similar clinical presentation. Through the certainty of clinical practice, it is more challenging to make a clear-cut diagnosis of endocarditis. The overdiagnosis or underdiagnosis of endocarditis in laboratory findings is required to avoid misdiagnosis. History physical examination and echocardiography are some of the careful analyzing properties to keep in mind. The identification of endocarditis is made in two phases: first initial assessment and therapy, and in the second phase, definitive diagnosis and treatment [24].

The Bentall process is a cardiac surgery associated with the graft replacing of the aortic valve, aortic root, and ascending aorta. After Bentall's process, graft infection is severe and much more challenging to diagnose and treat. PET/CT is an alternative method for diagnosis. If hypodense accumulation observes in the periaortic graft, which confirms the diagnosis, the surgery remains the desirable treatment option. However, it may not always be feasible, given the complexity and high-risk nature of the procedure. Medical therapy with prolonged antibiotics is effective for non-surgical candidates. PET/CT correlates poorly with the clinical response; hence, it is not suggested at follow-up [25].

Antimicrobial therapy

The main focus of antimicrobial therapy is to avoid toxicity to the host and the emergence of resistance. It is not just limited to the gram-positive organism but the microbial spectrum [26]. The risks and caveats are the factors created by the extended use of antibiotic therapy. The properties of antimicrobial therapy are pharmacodynamics, pharmacokinetics, and antimicrobial agents. Clinicians maximize the antimicrobial treatment in infective endocarditis, which is the primary clinical consideration. The parenteral antibiotics have a specific period of action of two to six weeks in the therapy. The uncomplicated *viridians* *group* *streptococci* species indicated that outpatient parenteral antibiotic treatment is much safer. The native infection causing endocarditis is *S. aureus*. The disease caused by the *S. aureus* species' primary therapy for management is rifampin [27].

The safety and efficacy of the treatment with rifampin native valve are not addressed in past studies. The advice for infection of hepatotoxicity, drug interactions, and the outgrowth of immune *S. aureus* are standard. Rifampin is a medication used to manage and treat various mycobacterial and gram-positive bacterial infections. The effective management of the native valve *S. aureus* infection requires an appropriate course of antimicrobial agents. One of every six infective endocarditis patients does not survive. Approximately 80 percent of patients can survive the initial hospitalization. In the infection with highly virulent organisms, one-third of patients may die as the indirect or direct result of valvular disease. Modern diagnostic techniques urge unfavorable outcomes in patients receiving appropriate antimicrobial therapy. The issues of frequent and inappropriate use of antibiotics, lack of educational awareness, ethics regarding antibiotic usage, and the lack of infection control and sanitation make the situation worse. The outpatient parenteral antibiotic therapy (OPAT) in the home environment and deemed sufficiently stable are comparable properties with the patients under treatment in the hospital. Intravenous antibiotics administration and hospitalization are the high cost in treating infective endocarditis. The vital success of OPAT in infective endocarditis is the careful selection of the patients. The patients will be hemodynamically stable without any clinical complications. Thoracic aortic prosthetic graft infection is relatively rare; patients undergoing thoracic aortic surgery have become older and feature more comorbidities. Experimental models are urgently needed to modify strategies for short- and long-term prevention of thoracic aortic graft infection [25-28].

The goals of OPAT are to allow patients to complete treatment safely and effectively. The home-based system included an infectious diseases physician, a microbiologist, a cardiologist, and a nurse disciplined in intravenous procedures. Outpatient treatment of infective endocarditis is still uncommon. At the same time, home intravenous antibiotic therapy is increasing for the various infectious diseases among microbial agents. Infective endocarditis present in a patient has a diversity of pathogens. The high susceptibility penicillin is advisable for treating *streptococci* infections, as the American Heart Association (AHA) reported. The native valve involved *Erysipelothrix rhsiopathee*, *enterobacter species*, *Enterococcus *species, *Staphylococcus *species, and *Streptococcus *species. These are some of the causative organisms.

Management and complications

The extracardiac and the adjacent heart structures are the few affected complications of infective endocarditis. The person with the infection has a higher risk of having congestive heart failure, the most severe complication, which leads to death [29]. Patients with heart failure require intensive medical care, and cardiac valve exchange should consider antimicrobial therapy irrespective of the duration. All the subjects with bacterial infective endocarditis are hemodynamically stable with no multiple large emboli. These may acquire at least one course of antimicrobial therapy to sterilize the septic valve earlier than the cardiac valve substitution reasoned.

The technological boundary of 2D echocardiography of infective endocarditis with valve vegetation is incontestable. It does not give adequate information to uphold the cardiac valve substitution entirely on echocardiographic collection. The heart failure mortality rate is higher than that in cardiac valve replacement surgery due to endocarditis infection. Aortic regurgitation and myocardial abscess are significant problems. Most of the patients may present with a variety of complications [29,30]. Management of infected prosthetic aortic grafts in the ascending and root is complex and multifaceted.

The rate of particular complications depends upon the variant of the infecting pathogen, the period of disease before therapy, and the type of treatment. Nevertheless, measuring the actual frequency of complications is much more challenging because the studies are founded on frequent retrospective charts and various diagnostic criteria. The cardiac and extracardiac are the two main categories of infective endocarditis. *Staphylococcus epidermidis* causes prosthetic valve endocarditis. Gram-negative bacteria are resistant to the most available antibiotics. The valve-related problems or the factors such as the position, types, and size are further analyzed in the prosthetic valve endocarditis; the changed valve should be examined well, per the retrospective study [31].

## Conclusions

Infective endocarditis is the fourth leading cause of life-threatening infectious disease syndrome. The advancement in antimicrobial therapy and the development of better diagnostic and surgical techniques have decreased morbidity and mortality. Using newfound clinical criteria and emphasizing echocardiography will guide the practitioner in correctly diagnosing the disease. The ventricular walls have papillary muscles that support the atrioventricular valves through the chordae tendineae. It is categorized into infective and non-infective endocarditis based on the presence of vegetation. In non-infective endocarditis, tiny and sterile vegetations aggregate cluster along the edges of the valves or cusps. This form of endocarditis is also called marantic endocarditis. In infective endocarditis, the body's result is inflammation, which occurs when the condition is transient. Bacteremia is the simple presence of bacteria, while septicemia is the presence and multiplication of bacteria in the blood. Pyemia (or pyaemia) is a type of sepsis that leads to widespread abscesses of a metastatic nature. Since the vegetations in endocarditis are typically friable, these are to be included and dislodged in the rapid stream of the blood, which gives rise to embolism and causes excessive and severe cardiac complications. Other rare forms of infective endocarditis are tuberculous, syphilitic, fungal, and viral endocarditis. The lesions on right-sided infection, left-sided pulmonary disease, and tricuspid valves are addressed deeply. The treatment given for infective endocarditis is antimicrobial therapy. Management of this comes along with adopting a healthy lifestyle and staying fit.
